# What improves access to primary healthcare services in rural communities? A systematic review

**DOI:** 10.1186/s12875-022-01919-0

**Published:** 2022-12-06

**Authors:** Zemichael Gizaw, Tigist Astale, Getnet Mitike Kassie

**Affiliations:** 1 Department of Environmental and Occupational Health and Safety, Institute of Public Health, College of Medicine and Health Sciences, University of Gondar, Gondar, Ethiopia; 2 International Institute for Primary Health Care- Ethiopia, Ethiopian Public Health Institute, Addis Ababa, Ethiopia

**Keywords:** Primary healthcare, Access to PHC services, Rural communities, Key strategies to improve access to PHC services

## Abstract

**Background:**

To compile key strategies from the international experiences to improve access to primary healthcare (PHC) services in rural communities. Different innovative approaches have been practiced in different parts of the world to improve access to essential healthcare services in rural communities. Systematically collecting and combining best experiences all over the world is important to suggest effective strategies to improve access to healthcare in developing countries. Accordingly, this systematic review of literature was undertaken to identify key approaches from international experiences to enhance access to PHC services in rural communities.

**Methods:**

All published and unpublished qualitative and/or mixed method studies conducted to improvement access to PHC services were searched from MEDLINE, Scopus, Web of Science, WHO Global Health Library, and Google Scholar. Articles published other than English language, citations with no abstracts and/or full texts, and duplicate studies were excluded. We included all articles available in different electronic databases regardless of their publication years. We assessed the methodological quality of the included studies using mixed methods appraisal tool (MMAT) version 2018 to minimize the risk of bias. Data were extracted using JBI mixed methods data extraction form. Data were qualitatively analyzed using emergent thematic analysis approach to identify key concepts and coded them into related non-mutually exclusive themes.

**Results:**

Our analysis of 110 full-text articles resulted in ten key strategies to improve access to PHC services. Community health programs or community-directed interventions, school-based healthcare services, student-led healthcare services, outreach services or mobile clinics, family health program, empanelment, community health funding schemes, telemedicine, working with traditional healers, working with non-profit private sectors and non-governmental organizations including faith-based organizations are the key strategies identified from international experiences.

**Conclusion:**

This review identified key strategies from international experiences to improve access to PHC services in rural communities. These strategies can play roles in achieving universal health coverage and reducing disparities in health outcomes among rural communities and enabling them to get healthcare when and where they want.

**Supplementary Information:**

The online version contains supplementary material available at 10.1186/s12875-022-01919-0.

## Introduction

Universal health coverage (UHC) is used to provide expanding services to eliminate access barriers. Universal health coverage is defined by the world health organization (WHO) as access to key promotional, preventive, curative and rehabilitative health services for all at an affordable rate and ensuring equity in access. The term universal has been described as the State's legal obligation to provide healthcare to all its citizens, with particular attention to ensuring that all poor and excluded groups are included [1–3].

Strengthening primary healthcare (PHC) is the most comprehensive, reliable and productive approach to improving people's physical and mental wellbeing and social well-being, and that PHC is a pillar of a sustainable health system for UHC and health-related sustainable development goals [4, 5]. Despite tremendous progress over the last decades, there are still unaddressed health needs of people in all parts of the world [6, 7]. Many people, particularly the poor and people living in rural areas and those who are in vulnerable circumstances, face challenges to remain healthy [8].

Geographical and financial inaccessibility, inadequate funding, inconsistent medication supply and equipment and personnel shortages have left the reach, availability and effect of PHC services in many countries disappointingly limited [9, 10]. A recent Astana Declaration recognized those aspects of PHC need to be changed to adapt adequately to current and emerging threats to the healthcare system. This declaration discussed that implementation of a need-based, comprehensive, cost-effective, accessible, efficient and sustainable healthcare system is needed for disadvantaged and rural populations in more local and convenient settings to provide care when and where they want it [8].

Different innovative approaches have been practiced in different parts of the world to improve access to essential healthcare services in rural communities. Systematically collecting and combining best experiences all over the world is important to suggest effective strategies to improve access to healthcare in developing countries. Accordingly, this systematic review of literature was undertaken to identify key approaches from international experiences to enhance access to PHC services in rural communities. The findings of this systematic literature review can be used by healthcare professionals, researchers and policy makers to improve healthcare service delivery in rural communities.

## Methodology

### Research question

What improves access to PHC services in rural communities? We used the PICO (population, issue/intervention, comparison/contrast, and outcome) construct to develop the search question [11]. The population is rural communities or remote communities in developing countries who have limited access to healthcare services. Moreover, we extended the population to developed countries to capture experiences of both developing and developed countries. The issue/intervention is implementation of different community-based health interventions to access to essential healthcare services. In this systematic review, we focused on PHC health services, mainly essential or basic healthcare services, community or public health services, and health promotion or health education. Primary healthcare is “a health care system that addressed social, economic, and political causes of poor health promotes health though health services at the primary care level enhances health of the community” [12]. Comparison/contrast is not appropriate for this review. The outcome is improved access to essential healthcare services.

### Outcome measures

The outcome of this review is access to PHC services, such as preventive, promotive, curative, rehabilitative, and palliative health services which are affordable, convenient or acceptable, and available to all who need care.

### Criteria for considering studies for this review

All published and unpublished qualitative and/or mixed method studies conducted to improve access to PHC services were included. Government and international or national organizations reports were also included. Different organizations whose primary mission is health or promotion of community health were selected. We included articles based on these eligibility criteria: context or scope of studies (access to PHC services), article type (primary studies), and publication language (English). Articles published other than English language, citations with no abstracts and/or full texts, reviews, and duplicate studies were excluded. We included all articles available in different electronic databases regardless of their publication years. We didn’t use time of publication for screening.

### Information sources and search strategy

We searched relevant articles from MEDLINE, Scopus, Web of Science, WHO Global Health Library, and Google Scholar to access all forms of evidence. An initial search of MEDLINE was undertaken followed by analysis of the text words contained in the title and abstract, and of the index terms used to describe articles. We used the aforementioned performance indicators of PHC delivery and the PICO as we described above to choose keywords. A second search using all identified keywords and index terms was undertaken across all included databases. Thirdly, references of all identified articles were searched to get additional studies. The full electronic search strategy for MEDLINE, a major database we used for this review is included as a supplementary file (Additional file 1: Appendix 1).

### Study selection and assessment of methodological quality

Search results from different electronic databases were exported to Endnote reference manager version 7 to remove duplication. Two independent reviewers (ZG and BA) screened out records. An initial screening of titles and abstracts was done based on the PICO criteria and language of publication. Secondary screening of full-text papers was done for studies we included at the initial screening phase. We further investigated and assessed records included in the full-text articles against the inclusion and exclusion criteria. We sat together and discussed the eligibility assessment. The interrater agreement was 90%. We resolved disagreements by consensus for points we had different rating. We used the PRISMA flow diagram to summarize the study selection processes.

Methodological quality of the included studies was assessed using mixed methods appraisal tool (MMAT) version 2018 [13]. As it is clearly indicated in the user guide of the MMAT tool, it is discouraged to calculate an overall score from the ratings of each criterion. Instead, it is advised to provide a more detailed presentation of the ratings of each criterion to better inform quality of the included studies. The rating of each criterion was, therefore, done as per the detail explanations included in the guideline. Almost all the included full text articles fulfilled the criteria and all the included full text articles were found to be better quality.

### Data extraction

We independently extracted data from papers included in the review using JBI mixed methods data extraction form. This form is only used for reviews that follow a convergent integrated approach, i.e. integration of qualitative data and qualitative data [14]. The data extraction form was piloted on randomly selected papers and modified accordingly. One reviewer extracted the data from the included studies and the second reviewer checked the extracted data. Disagreements were resolved by discussion between the two reviewers. Information was extracted from each included study on: list of authors, year of publication, study area, population of interest, study type, methods, focus of the studies, main findings, authors’ conclusion, and limitations of the study.

### Synthesis of findings

The included full-text articles were qualitatively analyzed using emergent thematic analysis approach to identify key concepts and coded them into related non-mutually exclusive themes. Themes are strategies mentioned or discussed in the included records to improve access to PHC services. Themes were identified manually by reading the included records again and again. We then synthesized each theme by comparing the discussion and conclusion of the included articles.

### Systematic review registration number

The protocol of this review is registered in PROSPERO (the registration number is: CRD42019132592) to avoid unplanned duplication and to enable comparison of reported review methods with what was planned in the protocol. It is available at https://www.crd.york.ac.uk/prospero/display_record.php?ID=CRD42019132592.

### Schematic of the systematic review and reporting of the search

We used PRISMA (Preferred Reporting Items for Systematic Reviews and Meta-Analyses) 2009 checklist [15] for reporting of this systematic review.

## Results

### Study selection

**Fig. 1:**
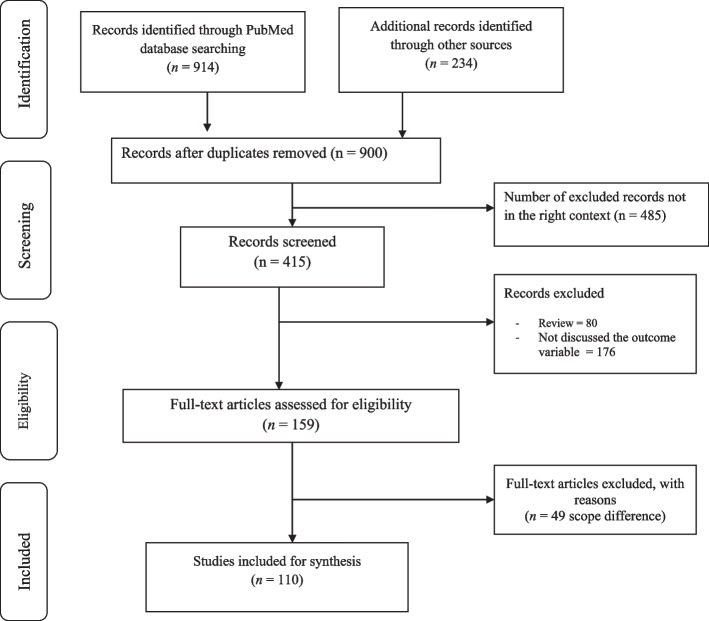
Study selection flow diagram

The search strategy identified 1148 titles and abstracts [914 from PubMed (Table 1) and 234 from other sources] as of 10 March 2022. We obtained 900 after we removed duplicated articles. Following assessment by title and abstract, 485 records were excluded because these records did not meet the criteria as mentioned in the method section. Additional 256 records were discarded because the records did not discuss the outcome of interest well and some records were systematic reviews. The full text of the remaining 159 records was examined in more detail. It appeared that 49 studies did not meet the inclusion criteria as described in the method section. One hundred ten records met the inclusion criteria and were included in the systematic review or synthesis (Fig. 1).

**Table 1:** Search terms and number of articles found in PubMed Advanced search as of 10 March 2022

Searches	Search term	Items found
01	“Rural communities”[All Fields]	8,220
02	“Remote population”[All Fields]	84
03	“Hard to reach areas”[All Fields]	203
04	“Medically underserved population”[All Fields]	8,394
05	1 or 2 or 3 or 4	16,648
06	“Healthcare service”[All Fields]	2,435
07	“Primary healthcare”[All Fields]	8,556
08	“Essential health services”[All Fields]	307
09	“Basic health services”[All Fields]	382
10	“Health extension program”[All Fields]	101
11	“Community health program”[All Fields]	225
12	“Health post”[All Fields]	582
13	“Community health worker” [All Fields]	1,576
14	“Strategies to improve healthcare” [All Fields]	24
15	“Access to essential healthcare services” [All Fields]	12
16	“Access to basic healthcare services” [All Fields]	7
17	“Equity to healthcare services” [All Fields]	30
18	“Health financing” [All Fields]	43
19	“Primary healthcare service delivery” [All Fields]	6
20	“Population health management” [All Fields]	51
21	“Availability of effective PHC services” [All Fields]	130
22	“Effective service coverage” [All Fields]	65
23	“Strategies to tackle healthcare barriers” [All Fields]	147
24	“Strategies to improve healthcare system” [All Fields]	36,735
25	6 or 7 or 8 or 9 or 10 or 11 or 12 or 13 or 14 or 15 or 16 or 17 or 18 or 19 or 20 or 21 or 22 or 23 or 24	66,124
26	5 and 25	914

**Fig. 2:**
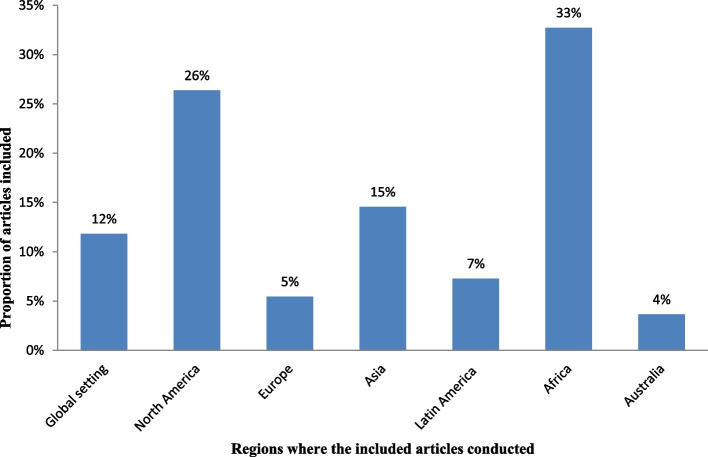
Regions where the included full-test articles conducted

Of 900 articles resulting from the search term, 110 (12.2%) met the inclusion criteria. The included full-text articles were published between 1993 and 2021. Ninety-two (83.6%) of the included full-text articles were research articles, 5(4.5%) were technical reports, 3 (2.7%) were perspective, 4 (3.6%) was discussion paper, 3(2.7%) were dissertation or thesis, 2 (1.8%) were commentary, and 1 (0.9) was a book. Thirty-six (33%) and 29 (26%) of the included full-text articles were conducted in Africa and North America, respectively (Fig. 2).

### Key strategies identified

The analysis of 110 full-text articles resulted in 10 themes. The themes are key strategies to improve access to PHC services in rural communities. The key strategies identified are community health programs or community-directed healthcare interventions, school-based healthcare services, student-led healthcare services, outreach services or mobile clinics, family health program, empanelment, community health funding schemes, telemedicine, promoting the role of traditional medicine, working with non-profit private sectors and non-governmental organizations (NGOs) including faith-based organizations (Table 2).

**Table 2:** Key strategies identified to improve access to PHC services in rural communities

Key strategies	References
Community health programs or community-directed healthcare interventions	[16–39]
School-based primary healthcare	[40–48]
Student-led healthcare services	[49–53]
Outreach services or mobile clinics	[54–71]
Family health program	[72–75]
Empanelment	[76–79]
Community health funding schemes	[80–90]
Telemedicine	[91–103]
Integrative medicine	[104–110]
Working with non-profit private sectors and non-governmental organizations	[111–125]

### Description of strategies

- a. Community health programs or community-directed healthcare interventions

Twenty-four (21.8%) of the full-text articles included in this review discussed that community health programs (CHPs) or community-directed healthcare interventions are best strategies to provide basic health and medical care close to the community to increase access and coverage of essential health services. Community health programs are locally based health promotion, disease prevention, and treatment programs available typically to communities in need and community-directed intervention strategy is an approach in which communities themselves direct the planning and implementation of intervention delivery. Rural communities, especially, in developing countries have no access to healthcare facilities in the near distance and have less chance to receive healthcare from doctors, health officers, nurses or midwives. In response to this critical problems, many countries have been investing heavily in community based primary health care to bring services to rural and remote areas where most of the population lives. Community health programs include construction of health posts or community health centers close to the community and deployment of community health workers (CHWs), such as health extension workers, to reach-out every village, who play a prominent role as the gatekeepers of healthcare in rural communities. Community-directed healthcare intervention is an approach in which communities themselves direct the planning and implementation of healthcare interventions. Community participation remains crucial in the identification of health problems, planning or designing of health interventions and implementation of the interventions, which enhances need-based and demand-driven provision of health services while promoting sustainability and ownership (Additional file 2: Appendix 2, Table A1).

- b. School-based primary healthcare

In this review, 9 of 110 (8.2%) of the included full-text articles pointed out that school-based healthcare services can be effective to improve access to PHC services. School-based health services are health programs that offer health care to children and youth either in a school or on school grounds and usually staffed according to school community needs and resources. School-based health services provide a variety of healthcare services to underserved children, youth and vulnerable populations in a convenient and accessible environment. Access to comprehensive health services via schools leads to improved access to healthcare (Additional file 3: Appendix 3, Table A2).

- c. Student-led healthcare services

In this review, 5 of 110 (4.5%) of the full-text articles discussed that the use of medical and health science students as healthcare service providers can minimize problems related with shortage of health professionals in rural healthcare system and can play appreciable roles to minimize healthcare service access problems in rural communities. Student-led healthcare services are developed through consultation between universities and local health providers and are purposefully designed clinical placements with a focus on clinical educational activities for pre-registration students. Student-led clinics link students, healthcare professionals, community-based organizations, universities, and communities. In this approach, students can gain practical experience in an interdisciplinary setting and through exposure to a community with unique and severe needs (Additional file 4: Appendix 4, Table A3).

- d. Outreach services or mobile clinics

In this systematic literature review, 18 of 110 (16.4%) of the included studies discussed that outreach services or mobile clinics in primary care and rural hospital settings can improve access to PHC services in rural communities. Mobile outreach service is defined as healthcare services provided by a mobile team of trained providers, from a higher-level health facility to a lower-level health facilities or locally available community facilities that are not used for clinical services, such as schools, health posts, or other community structures. Outreach services improve access to specialists and hospital-based services, strengthen connections between specialists and PHC providers, and give the benefits of consultations in primary care settings. Specialist outreach services have the potential to overcome access barriers faced by disadvantaged rural and remote communities. Furthermore, a community-based mobile clinics can be effective in uncovering illness and in directing patients to a healthcare home (Additional file 5: Appendix 5, Table A4).

- e. Family health program

Four (3.6%) of the included full-text articles discussed that family health program (FHP) is highly cost-effective tool for improving access to healthcare services for deprived areas (such as rural communities). Family health program means the program is a program designed to provide primary care as well as the prevention and early treatment of communicable and non-communicable diseases in defined populations by deploying interdisciplinary healthcare teams include physicians, nurses, nurse assistants, and full-time community health agents. It has evolved into a robust approach to providing primary care for defined populations by deploying interdisciplinary healthcare teams. The nucleus of each team includes a physician, a nurse, a nurse assistant, and full-time community health agents. This approach is effective on improving access to healthcare and eliminating health disparities (Additional file 6: Appendix 6, Table A5).

- f. Empanelment

This systematic review of literature identified that empanelment (also known as rostering) is a best strategy to proactively provide coordinated primary healthcare towards achieving universal health coverage. Empanelment is a continuous, iterative set of processes that identify and assign populations to facilities, care teams, or primary care providers who have a responsibility to know their assigned population. It enables health systems to improve health outcomes and to reduce costs. Empanelment establishes a point of care for individuals and simultaneously holds primary healthcare providers and care teams accountable for actively managing care for a specific group of individuals (Additional file 7: Appendix 7, Table A6).

- g. Community health funding schemes

In this systematic review of literature, 11 (10%) of the included articles discussed that community health funding schemes such as community-based health insurance (CBHI) increases access to healthcare services in low-income rural communities. Community-based health insurance schemes are usually voluntary and characterized by community members pooling funds to offset the cost of healthcare. Moreover, this approach is effective to mobilize domestic resources for health at low income levels. For low-income countries, community health financing has modest ability to increase the total amount of funds for healthcare. Properly structured community health financing system can significantly improve efficiency, reduce the cost of healthcare, improve quality and health outcomes, and pool risks. Community-financing schemes could improve preventive services and reduce the incidence of diseases. It could also improve people’s access to healthcare and the quality of services, thus improving their health status. Community health financing could also improve risk pooling and reduce health-induced impoverishment. Community health insurance has potential positive impacts on health and social security (Additional file 8: Appendix 8, Table A7).

- h. Telemedicine

In this review, 13 of 110 (11.8%) articles discussed that telemedicine is one of the solutions for rural subspecialty healthcare delivery. Telemedicine can be defined as the use of technology (computers, video, phone, messaging) by a medical professional to diagnose and treat patients in a remote location. The provision of subspecialty services using telemedicine to a remote and medically underserved population provides improved access to subspecialty care. Telemedicine brings sustainable healthcare to rural populations. Use of information and communication technologies in support of health and health-related fields, including healthcare services, health surveillance, health education, and health research has the potential to greatly improve health service efficiency, expand or scale up treatment delivery to thousands of patients in the rural populations (Additional file 9: Appendix 9, Table A8).

- i. Promoting the role of traditional medicine

Seven (6.4%) of the included articles showed that incorporating traditional healers into public health system addresses healthcare needs of people with limited access to allopathic medicine. Traditional medicine is the sum total of the knowledge, skill, and practices based on the theories, beliefs, and experiences indigenous to different cultures, whether explicable or not, used in the maintenance of health as well as in the prevention, diagnosis, improvement or treatment of physical and mental illness. Knowledge about traditional medicine has a catalyzing effect in meeting health sector development objectives. Integrating traditional medicine into national health systems in combination with national policy and regulation for products, practices and providers can enhance access to PHC services in remote populations (Additional file 10: Appendix 10, Table A9).

- j. Working with non-profit private sectors and non-governmental organizations

In this systematic review, 15 of 110 (13.6%) of the included articles revealed that working with non-profit private sectors and NGOs strengthens the healthcare system. Involving the non-profit private sectors, faith-based organizations (FBOs), and NGOs for health system strengthening eventually contributes to create a healthcare system reflecting an increased efficiency, more equity and good governance in health. International and local NGOs have endeavored to fill the gaps in access to healthcare services, research and advocacy. Non-profit private sectors and NGOs have a key role in improving health in low- and middle-income countries. With networks that reach even the most remote communities, many FBOs are well positioned to promote demand and access for healthcare services. Partnership among FBOs is critical in increasing access to healthcare services, and ensuring sustainability by influencing behaviors at the community, family and individual level. Faith-based organizations play an integral role in the healthcare system by increasing health seeking behaviors and delivering supportive services that address common access and cultural barriers (Additional file 11: Appendix 11, Table A10).

## Discussion

This systematic literature review found that community health programs or community-directed healthcare interventions, school-based healthcare services, student-led healthcare services, outreach services or mobile clinics, family health program, empanelment, community health funding schemes, telehealth, integrative medicine, and working with non-profit private sectors and NGOs are key strategies to improve access to PHC services in rural communities. The identified strategies address the four major pillars of primary healthcare (i.e., community participation, inter-sectoral coordination, appropriate technology, and support mechanism made available) [126]. Moreover, the identified strategies are effective to improve access to healthcare services to rural communities. Moreover, the identified strategies are effective to solve shortage of manpower and to build knowledge and skill of the local health workforces in rural healthcare system. The ability of a healthcare system to meet health needs of the population depends largely on the knowledge, skills, motivation and deployment of the people responsible for organizing and delivering health services. The results of this review can strengthen the health information system, which are core elements of the healthcare system that ensure community engagement through dissemination and use of timely and reliable health information to rural populations. This review also suggests strategies to narrow down the health disparities among rural populations, which is wide in most Least and Middle Income Countries (LMICs). Healthcare services are usually disproportionately concentrated in major urban areas. As a result, rural communities face growing health disparities, largely attributed to weak policies, inefficiencies, poor leadership, and governance in healthcare system.

This review identified that community health programs or community-directed healthcare interventions address health disparities by ensuring equitable access to health resources in communities where health equity is limited by socioeconomic and geographical factors. Community health programs include identifying and prioritizing public health problems in a specific geographic area; designing and implementing public health interventions (such as establishing community health centers, mobile clinics, and outreach programs); providing services (such as health education, screenings, social support, and counseling), and deploying community health workers to promote healthy behaviors; advocating for improved care for populations at risk; and working with stakeholders to address community healthcare needs [16–18, 127–130]. The community-oriented PHC model which is socially responsive medicine makes a healthcare system more rational, accountable, appropriate, and socially relevant to the public. Consequently, this model serves as a paradigm for reforming healthcare systems. Community-directed interventions can be considered as a realistic means to increase accessibility of interventions at community-level in rural areas [32–38]. This approach is best in situations where there are cultural barriers to implement interventions because this strategy is effective to develop ownership in the community. In-service and on-the-job training for community health workers, close supervision and government support, and program evaluation is very important to strengthen the community health program [131–133].

This review identified that school-based PHC services are effective strategies to improve access to PHC services. School-based health services provide a variety of healthcare services to children, youth and vulnerable populations in a convenient and accessible environment which indirectly improve leadership and governance. Science teachers and home room teachers play important roles to implement this strategy. It impacts on delivering preventive care such as immunizations, managing chronic illnesses and providing reproductive health services for adolescents. Comprehensive health services via schools improve access to healthcare information [40–47]. Access to school around the world increased drastically in the last century [134]. This high schooling rate is a good opportunity to provide healthcare services to school learners in accessible places and to disseminate health messages to families. Prior researches suggest that school-based healthcare services increase access to healthcare by increasing utilization of primary care, prevention services, and health maintenance visits [135, 136]. Including science teachers, home room teachers, school principals, students, communities, community health workers, and other interested parties in the school-based healthcare system as main actors or promoters must be considered to sustain the impact. Health and education sectors should work in collaboration with the above-mentioned actors to plan, implement and monitor the progress. School-based healthcare services are preferable in situations when there is high schooling rate and limited access to healthcare institutions. This strategy is also an alternative way in areas where the health seeking behavior of the community is low.

The use of medical and health science students in rural healthcare system was identified as a key strategy to minimize health inequalities in rural communities due to shortages in health workforce and distribution of healthcare resources [49–53]. Student-led health intervention is an alternative approach to provide essential healthcare services to the community where there is shortage of healthcare workers [137, 138]. Students will have opportunities to learn professional skills and competencies while they are providing healthcare services to the community. Moreover, benefits for student learning include increased communication, collaboration, and leadership skills [53, 139]. Student-led health intervention also enables increased access to services, more time for assessments and treatments, increased depth of health teaching, holistic and integrated healthcare, and free health supports [140–143]. However, the use of medical and health science students in the rural healthcare system may have ethical and competency issues. Supporting strategies such as close supervision, preparing clear protocols, and including senior experts in the team should be considered.

This systematic review of literature found that outreach services or mobile clinics can improve access to PHC service delivery in rural populations [54–69]. In developing countries, the highest proportion of people lives in rural areas where doctor services are not available. Rural communities travel to major cities to get specialist services. This reflects a desire for closer integration between primary and secondary care. Specialist outreach services or mobile clinics have become one of the effective solution to solve health disparities, to improve access to healthcare services, and to build capacity of local healthcare workforces. This strategy is preferable in situations when there are high loads in tertiary or referral level hospitals and when there is high patient leakage in the referral system [63–69]. However, the implementation may not be easy. It needs well established healthcare system and budget. Moreover, the efficiency of care may be lower compared with hospital-based cares and the effect on patients’ health outcomes might be small [56, 57, 61]**.** Irregular specialist visits in rural areas may not have real impacts unless the services are sustainable with a strong commitment at national and local levels. Outreach activities should be included in health policies with strong leadership, healthcare financing, and private initiatives must be encouraged to maintain the activities over time.

This review revealed that FHP is highly effective tool for improving health for rural communities. The FHP has provided a new, more robust model of primary healthcare services designed to provide accessible, first contact, comprehensive, and whole person care that is coordinated with other healthcare services. It has positive results to improved availability, access to, and use of health services, and improved health indicators, such as reduced infant mortality, improved detection of cases of neglected diseases, and reduced health disparities [73, 144–146]. The FHP deploys interdisciplinary healthcare teams. The team includes a physician, a nurse, a nurse assistant, and full-time community health agents. Family health teams are organized geographically. The teams are responsible for delivering public health interventions [72, 74]. Family health program is an alternative strategy in rural healthcare system in situations when there are inequities in access to care; when there is high hospitalization rate; when there is low health seeking behavior in the community; and when there is poor case detecting and reporting system. Despite these remarkable achievements, the FHP has some challenges include difficulties in the recruitment and retention of doctors trained appropriately to deliver primary healthcare, large variations in quality of local care, patchy integration of primary care services with existing secondary and tertiary care, and slow adoption of FHP in large population [147].

In this review, empanelment has been identified as a best strategy to deliver coordinated primary healthcare towards achieving universal health coverage [76–79]. The goal of empanelment is provide people-centered healthcare services based on their needs to ensure that every established patient receives optimal care, whether he/she regularly visits healthcare centers. Major activities in this approach include assignment of all patients to a healthcare provider panel; update panel assignments on a regular basis; and use panel data to educate, and track patients [79]. Empanelment enables healthcare systems to improve patient experiences, reduce costs, and improve health outcomes. Empanelment is an effective strategy to deliver four key functions: first-contact accessibility, continuity, comprehensiveness, and coordination [148]. Effective empanelment requires responsibility for the health of a target population, including providing healthcare services based on their health status, which is an important step in moving towards people-centered integrated healthcare [79].

This review identified that community health funding schemes such as community-based health insurance (CBHI) increases access to healthcare in low-income rural communities. Moreover, this approach is effective to mobilize domestic resources for health at low income levels [80–90]. Community-based health insurance is an emerging strategy to provide financial protection against the cost of illness. It is an effective strategy to improve access to quality health services for low-income rural households [149]. Existence of social capital in the community is a determinant factor for the effectiveness of CBHI as social capital has a positive effect on the community's demand for insurance [150, 151]. Moreover, solidarity and trust between the members are the key principles for the good functioning of a CBHI. Solidarity and trust stir-up members who are susceptible to risk to put together their resources for common use [149, 152, 153]. Affordability of premiums or contributions, technical arrangements made by the scheme management, timing of collecting the contributions, trust in the integrity and competence of the managers of the CBHI, The quality of care offered through the CBHI, accessible across different population groups are some of the determinant factors to be considered to increase people’s decision to join the CBHI schemes [154, 155].

In this review, telemedicine has been identified as one of the many possible solutions for rural subspecialty healthcare delivery. Telemedicine is a vital technological tool to increase healthcare access, improve care delivery systems, engage in culturally competent outreach, health workforce development, and health information system [91–100]. Telemedicine can be a great alternative to the traditional healthcare system in situations like diagnoses of common medical problems; inquiries about various medical issues for home treatments; post-treatment check-ins or follow-up for chronic care; holidays, weekends, late night or any other situation when regular medical care is not possible; patient inability to leave the house; patients who lack regular access to relevant medical expertise in their geographic area*;* and etc. However, technological issues are challenges when dealing with telemedicine, especially in developing countries. General problems of Internet connectivity and access to infrastructure can minimize benefits of this strategy. Costs associated with technology can also be a barrier. Furthermore, health technology requires human capacity to use it. Therefore, strengthening the information communication technologies (ICT) and human capacity building on ICT are important to address the health needs of the rural communities.

This systematic review of literature identified that promoting the role of TM solves problems of access to allopathic medicine. Integration of TM in health system will result in increased coverage and access to healthcare services. The role of complementary and alternative medicine for health is undisputed particularly in light of its role in health promotion and well-being. It also supports local health workforces [104–109]. Incorporating traditional healers into the public health system addresses healthcare needs [156, 157]. However, integrating TM to the public healthcare system is challenging. It is a general belief that TM defies scientific procedures in terms of objectivity, measurement, codification and classification [157]. If integrated, who provides training to medical doctors on the ontology, epistemology and the efficacies of TM in modern medicine [157]. Due to these, some scholars suggest that both TM and modern medicine be allowed to operate and develop independent of one another [158, 159]. Another fundamental challenge to TM is the widespread reported cases of fake healers and healings [157]. Generally, this strategy is more of feasible in areas where formal trainings on integrative medicine are available. Even though the integration is challenging, the health sector can use traditional healers as health educators or health promoters by providing training and continuous support. It can be also possible to use traditional healers as facilitators in the community-directed approaches. In general TM can be used in the primary healthcare system where no access to allopathic medicine and when conventional medicine is ineffective in treatment of disease [160].

Working with non-profit private sectors and NGOs has been identified as effective strategies to strengthen the healthcare system in developing countries [111–118]. Since governments in developing countries are challenged to meet the health needs of their populations because of financial constraints, limited human resources, and weak health infrastructure; the private sector (especially the non-profit private sectors) and non-governmental organizations can help expand access to healthcare services through its resources, expertise, and infrastructure. However, the presence of an NGO in the operation, may contribute to unrealistic expectations of health services, affecting perceptions of the latter negatively [113]. Moreover, reports have it that besides other issues in many instances NGOs allocated funds only to disease specific projects (vertical programming) rather than to broad based investments (horizontal programming) [161]. There are also concerns that donor expenditures in developing countries are not only unsustainable but may be considered as inadequate considering the enormous healthcare burden [161–164]. To avoid unrealistic expectations and dissatisfaction, and to increase and sustain the population’s trust in the organization, NGOs should operate in a manner that is as integrated as possible within the existing structure and should work close to the population it serves, with services anchored in the community. Moreover, faith-based organizations contribute in health such as disease prevention, health education or promotion, and community health development beyond psychological and spiritual care [119–124]. Religious organizations can reach all segments of rural populations. Therefore, integrating PHC services, especially health education and promotion, diseases prevention and community health development with religious organizations intensifies delivery of healthcare services. Working with FBOs is a best way in situations where cultural and faith-based barriers are common and in areas, where access problems are often related to lack of providers. However, religious organizations need intensive training on health promotion and health system to enable them to respond to local contexts within the framework of national policies. Moreover, there should be strong partnership with government agenesis to sustain the effort [165–168].

### Contribution of this review

Various studies reported one or more strategies to improve access to primary healthcare services. However, the strategies reported by individual studies are not compiled together and there is lack of pooled evidence on effective strategies to improve access to healthcare system. This systematic literature review was, therefore, conducted to compile effective strategies to improve access to healthcare services in rural communities. The review suggests key strategies to improve access to PHC services in rural communities. These suggested strategies are implementable in countries that suffer from shortage of health workers and healthcare financing because all the strategies used locally available opportunities. The local healthcare system needs, therefore, scan the available opportunities in the locality for implementing the suggested strategies and needs to integrate the strategies in the healthcare system to sustain the impacts. Healthcare providers, researchers and policy makers could use the results of this systematic literature review to increase access to healthcare services in hard-to-reach areas. As the strategies are compiled from experiences of different countries (developed and least developed countries), there might be contextual differences like socio-economic, cultural, institutional, and geographical challenges to adopt the identified strategies. Moreover, some of the experiences only come from one or two countries. Therefore, strategy developers and implementers need to consider these contextual challenges or variation during adopting and implementing different strategies.

### Strengths and limitations of the study

As a strength, this systematic review explores international (both developed and developing countries) best experiences on primary healthcare service delivery and identified ten key approaches to improve access to PHC services in rural communities. We also searched relevant published or unpublished articles, dissertations or theses, discussion papers, and perspectives from a wide range of sources, such as MEDLINE, Scopus, Web of Science, WHO Global Health Library, and Google Scholar.

As a limitation, we entirely relied on electronic databases to search relevant articles. We didn’t include locally available printed out records. We also applied limits for language. We excluded articles published other than English language. We believed we could get more relevant articles if we had access to records available in prints and if we include articles published other than English language. Furthermore, since the strategies are compiled from experiences of different countries (developed and least developed countries), there might be contextual differences like socio-economic, cultural, institutional and geographical challenges to adopt the identified strategies. There was also limited evidence for some articles, especially reports to rate their methodological quality. Readers should also note that our review might missed some important work in improving access to PHC services and the identified strategies are not the only strategies to improve access to PHC services. There might be other effective strategies which are not included in this review. In addition generalizability might be affected since some of the experiences only come from one or two countries. Moreover, this review focuses on access not quality of care delivered.

## Conclusion

This review identified key strategies from international experiences to improve access to PHC services in rural communities. These strategies are effective to improve access to healthcare services in rural or remote communities. They can also play roles in achieving UHC and reducing disparities in health outcomes and increase access to rural communities to get healthcare when and where they want. Therefore, incorporating these key strategies suggested by this review in to the healthcare system is useful to enhance PHC services and to minimize impacts of health disparity in rural communities. However, the identified strategies may not be easy to implement. Increasing number and capacity of human resource for health; strengthening the healthcare financing system; improving medicine and supplies; working in different partners and communities; establishing monitoring and evaluation system; strong and committed leadership; and encouraging private initiatives must be considered to implement and maintain these strategies over time. Moreover, policy makers, program planners and implementers who want to utilize findings of this review should be aware that these are not the only effective strategies to improve access to primary healthcare services.

## Supplementary Information

**Additional file 1: **Searchstrategy. MEDLINE (PubMed).

**Additional file 2: Appendix 2: Table A1.**Description of full-text articles which discussed community health programs or community-directed interventions as a strategy to improve PHC service delivery in ruralcommunities.

**Additional file 3:** **Appendix 3: Table A2.**Description of full-text articles which discussed school-based healthcareservices as a strategy to improve PHCservice delivery in rural communities.

**Additional file 4:** **Appendix 4: Table A3.** Description of full-text articles which discussed student-led healthcareservices as a strategy to improve PHC service delivery in ruralcommunities.

**Additional file 5: Appendix 5: Table A4. **Descriptionof full-text articles which discussed outreach services or mobile clinics as astrategy to improve PHC service delivery in ruralcommunities.

**Additional file 6:** **Appendix 6: Table A5.**Description of full-text articles which discussed family health program as astrategy to improve PHC service delivery in rural,communities.

**Additional file 7:** **Appendix 7: Table A6.** Description of full-text articles whichdiscussed empanelment as a strategy to improve PHC service delivery in ruralcommunities.

**Additional file 8:** **Appendix 9: Table A8.**Description of full-text articles which discussed telemedicine or mobile healthas a strategy to improve PHC service delivery in ruralcommunities.

**Additional file 9:** **Appendix 8: Table A7.**Description of full-text articles which discussed community health funding schemes as a strategy to improve PHC service delivery in ruralcommunities.

**Additional file 10:** **Appendix 10: Table A9.**Description of full-text articles which discussed promoting the role of workingwith traditional healers as a strategy toimprove PHC service delivery in rural communities.

**Additional file 11:** **Appendix 11: Table A10.**Description of full-text articles which discussed working with non-profitprivate sectors and non-governmental organizations as a strategy to improve PHC service delivery in rural communities.

## Data Availability

All the extracted data are included in the manuscript.

## Abbreviations

CBHI: Community-based health insurance
FBOs: Faith-based organizations
FHP: Family health program
ICT: Information communication technologies
MMAT: Mixed methods appraisal tool
NGOs: Non-governmental organizations
PHC: Primary healthcare
PHCPI: Primary Health Care Performance Initiative
PICo: Population, phenomena of interest and context)
TM: Traditional medicine
UHC: Universal health coverage
