# Supplementary material for: What improves access to primary healthcare services in rural communities? A systematic review
Source: BMC Prim Care. 2022 Dec 6;23:313. doi: 10.1186/s12875-022-01919-0 (PMC9724256; doi:10.1186/s12875-022-01919-0)
Supplement: Supplementary file 1 — Additional file 1: Searchstrategy. MEDLINE (PubMed). [file 12875_2022_1919_MOESM1_ESM.docx]

**Search strategy: MEDLINE (PubMed)**

01. "Rural communities"[All Fields]

02. "Remote population"[All Fields]

03. "Hard to reach areas"[All Fields]

04. "Medically underserved population"[All Fields]

05. 1 or 2 or 3 or 4

06. "Healthcare service"[All Fields]

07. "Primary healthcare"[All Fields]

08. "Essential health services"[All Fields]

09. "Basic health services"[All Fields]

10. "Health extension program"[All Fields]

11. "Community health program"[All Fields]

12. "Health post"[All Fields]

13. “Community health worker” [All Fields]

14. "Strategies to improve healthcare" [All Fields]

15. "Access to essential healthcare services" [All Fields]

16. "Access to basic healthcare services" [All Fields]

17. "Equity to healthcare services" [All Fields]

18. " Health financing" [All Fields]

19. "Primary healthcare service delivery" [All Fields]

20. “Population health management” [All Fields]

21. “Availability of effective PHC services” [All Fields]

22. “Effective service coverage” [All Fields]

23. "Strategies to tackle healthcare barriers" [All Fields]

24. "Strategies to improve healthcare system" [All Fields]

25. 6 or 7 or 8 or 9 or 10 or 11 or 12 or 13 or 14 or 15 or 16 or 17 or 18 or 19 or 20 or 21 or 22 or 23 or 24

26. 5 and 25
