# Supplementary material for: What improves access to primary healthcare services in rural communities? A systematic review
Source: BMC Prim Care. 2022 Dec 6;23:313. doi: 10.1186/s12875-022-01919-0 (PMC9724256; doi:10.1186/s12875-022-01919-0)
Supplement: Supplementary file 2 — Additional file 2: Appendix 2: Table A1.Description of full-text articles which discussed community health programs or community-directed interventions as a strategy to improve PHC service delivery in ruralcommunities. [file 12875_2022_1919_MOESM2_ESM.docx]

Supplementary material Appendix 2 Table A1: Description of full-text articles which discussed community health programs or community-directed interventions as a strategy to improve PHC service delivery in rural communities

| Authors | Country | Article type | Findings |
| --- | --- | --- | --- |
| Admassie A, et al, 2009 | Ethiopia | Research article | The health extension program has significantly increased utilization of insecticide-treated bed nets and the proportion of children fully and individually vaccinated against tuberculosis, polio, diphtheria, pertussis, tetanus, and measles. |
| Arwal SH, 2015 | Afghanistan | Research article | Health posts provide health education and promotion, preventive care such as drug distribution and birth spacing devices, and limited curative services like diagnosis and treatment of common disease in children and adults, community mobilization, and referral of patients to health facilities. |
| Assefa Y, et al, 2019 | Ethiopia | Research article | The health extension program (HEP) enabled Ethiopia to achieve significant improvements in maternal and child health, communicable diseases, hygiene and sanitation, knowledge and healthcare seeking behavior. The program is also dynamic enough to shift tasks between health centers and the community. |
| Croke K, et al, 2020 | Ethiopia | Research article | Ethiopia’s facility construction program improved access to antenatal and delivery care. Opening of new health facilities within 5 km increases facility delivery by 7.2 percentage points (95% CI 5.2 to 9.1) and antenatal care by 0.38 visits (95% CI 0.24 to 0.52). |
| Datiko D & Lindtjørn B, 2009 | Ethiopia | Research article | Health extension workers improve tuberculosis case detection and treatment success in the rural communities in southern Ethiopia. |
| Feltner FJ, et al, 2012 | USA | Research article | This study examined effectiveness of a community health worker (CHW)–delivered cancer education program designed to increase knowledge and awareness of colorectal cancer screening options and found that CHWs are effective to increase knowledge and awareness about colorectal cancer screening education. |
| Hughes MM, et al, 2016 | USA | Research article | The Community Health Worker (CHW) model has been used to combat disparities in healthcare access by utilizing community members as healthcare liaisons to promote improved community health. CHW interventions, for instance, have been effective in improving diabetes management in the studied region. CHWs can conduct door-to-door outreach services. |
| le Roux KW, et al, 2020 | South Africa | Research article | Home visits by paraprofessional community health workers (CHWs) has been shown to improve maternal and child health outcomes in the rural communities. Home visiting has important effects on child health, maternal wellbeing and health behaviors. |
| Medhanyie A, et al, 2012 | Ethiopia | Research article | The health extension workers (HEWs) seem to have substantial contribution in several aspects of utilization of maternal health services, such as utilization of family planning, antenatal care and HIV testing. |
| Negussie A and Girma G, 2017 | Ethiopia | Research article | Health Posts are important health care delivery settings and their share from the overall service delivery of ANC, Family planning and child treatment services were pivotal. |
| Panday S, et al, 2017 | Nepal | Research article | Female community health volunteers (FCHVs) have potentials to deliver basic maternity care, promote health-seeking behavior so that serious delays in receiving healthcare can be minimized, share key health messages through regularly held mothers’ group meetings, and referred women for health checks. |
| Rahmawati R, & Bajorek B, 2015 | Indonesia | Research article | Community health workers (CHWs) played a prominent role as the gatekeepers of healthcare in rural communities. CHWs, for instance, facilitating blood pressure checks and physical exercise and providing health education. CHWs have potentials to liaise between rural communities and the healthcare system. |
| Sakeah E, et al, 2014 | Ghana | Research article | Community health office-midwives provide integrated services at the doorsteps of rural households that include skilled delivery to address the gap in skilled attendance. The integration of the skilled delivery program with the community-based health planning and services (CHPS) program appears to be an effective model for improving access to skilled birth attendance in rural communities. |
| Sarmento DR, 2014 | Timor-Leste | Research article | Integrating traditional birth attendance into a national healthcare system through family health promoter program has been programmatic effective. Traditional birth attendants have performed variety of tasks including outreach and case finding, health and patient education, referrals, home visits, and care management. |
| Than KK, et al, 2017 | Myanmar | Research article | Home births are common and auxiliary midwives were perceived as an essential care provider during childbirth in hard-to-reach areas. Main reasons provided were that auxiliary midwives are more accessible than midwives, live in the hard-to-reach areas, and are integrated in the community and well connected with midwives. |
| Yitayal M, et al, 2014 | Ethiopia | Research article | The health extension program (HEP) helps to increase utilization of contraceptives in rural community and improve family planning. Mothers from households which fully benefited from the HEP (model households) were 3.97 (adjusted odds ratio, 3.97; 95% confidence interval, 3.01–5.23) times more likely to use contraceptives compared with mothers from non-model households. |
| Braimah JA, et al, 2019 | Ghana | Research article | This study depicted that community-based health planning and service (CHPS) policy enhances access to primary health care (PHC) service. Findings of this study indicated that women who resided in CHPS zones (OR = 1.612; P ≤ 0.01) were more likely to have access to health care compared with their counterparts who resided in non-CHPS zones. |
| Brieger WR, et al, 2015 | Africa | Research article | Community-directed interventions have the potential for fulfilling the promise of primary health care by reaching underserved populations in various settings. Community-directed interventions have been successfully tested by expanding access to additional health services like malaria case management. |
| Katabarwa MN, et al, 2005 | Uganda | Research article | Community-directed interventions promote community involvement and integration of health care and developmental activities in an environment where both vertical and horizontal programs existed efficiently and effectively. |
| Madon S, et al, 2018 | Tanzania | Research article | Community participation is an effective strategy for developing sustainable village health governance. Community participation plays roles in sustaining health intervention programs and for sensitizing institutional and policy reform. |
| Makaula P, et al, 2012 | Malawi | Research article | Community-directed interventions are a realistic means to increase accessibility of health interventions at community level. Community-directed interventions intensify community participation to strengthen PHC, particularly within the areas of provision of insecticide treated bed nets, home case management for malaria, management of diarrhoeal diseases, treatment of schistosomiasis, and provision of food supplements against malnutrition. |
| Okeibunor JC, et al, 2011 | Nigeria | Research article | Community-based programs can substantially increase access to healthcare in general, and antenatal care attendance in particular in combination with supply side interventions. |
| Witmer A, et al, 1995 | USA | Research article | Community-oriented approach of community health workers is a key strategy for expanding access, reducing costs, and improving quality. Community health workers play important roles to function the health care system efficiently, encourage preventive and primary care, and accommodate previously underserved populations. |
| Wright RA, 1993 | USA | Research article | The community-oriented primary care model makes a healthcare system more rational, accountable, appropriate, and socially relevant to the public. Consequently, this model, may serve as a paradigm for reforming the organization and provision of healthcare services. |
