# Supplementary material for: What improves access to primary healthcare services in rural communities? A systematic review
Source: BMC Prim Care. 2022 Dec 6;23:313. doi: 10.1186/s12875-022-01919-0 (PMC9724256; doi:10.1186/s12875-022-01919-0)
Supplement: Supplementary file 3 — Additional file 3: Appendix 3: Table A2.Description of full-text articles which discussed school-based healthcareservices as a strategy to improve PHCservice delivery in rural communities. [file 12875_2022_1919_MOESM3_ESM.docx]

Supplementary material Appendix 3, Table A2: Description of full-text articles which discussed school-based healthcare services as a strategy to improve PHC service delivery in rural communities

| Authors | Country | Article type | Findings |
| --- | --- | --- | --- |
| Allison MA, et al, 2007 | USA | Research article | School-based health centers augment access to care and quality of care for underserved adolescents compared with traditional outpatient care sites. |
| Bozigar M, et al, 2020 | USA | Research article | School nurses may be a feasible strategy to overcome barriers to increasing healthcare services, for instance vaccination rates in medically underserved areas. Coordinating and sustaining partnerships between public health agencies, school districts, and school nurses are needed. |
| Brindis CD, et al, 2003 | USA | Research article | School-based healthcare service has become an increasingly acceptable source of medical care for children and adolescents. It demonstrates leadership by implementing medical standards of care and providing accountable source of healthcare. |
| Gibson EJ, et al, 2013 | USA | Research article | Access to comprehensive health services via schools leads to improved access to healthcare and improved quality of care. |
| Hutchinson P, et al, 2012 | USA | Research article | School-based healthcare services can increase the use of physical and behavioral health services and potentially decreasing the likelihood that adolescents will engage in risky behaviors. |
| Kaplan DW, et al, 1999 | USA | Research article | School-based healthcare services can be an effective component of health delivery system. It provides a better healthcare access and use to medically underserve and minority children. |
| Keeton V, et al, 2012 | USA | Research article | School-based health services provide a variety of healthcare services to children, youth and vulnerable populations in a convenient and accessible environment. It impacts on delivering preventive care, such as immunizations; managing chronic illnesses; providing reproductive health services for adolescents. |
| Minguez M, et al, 2015 | USA | Research article | School-based healthcare services can be an important access point to reproductive healthcare. Students with access to comprehensive reproductive health services via school-based healthcare services reported greater exposure to reproductive health education and counseling. |
| Paschall MJ, et al, 2018 | USA | Research article | School-based healthcare services increase availability of healthcare, such as mental health services that can help to reduce depressive episodes and suicide risk among adolescents. |
