# Supplementary material for: What improves access to primary healthcare services in rural communities? A systematic review
Source: BMC Prim Care. 2022 Dec 6;23:313. doi: 10.1186/s12875-022-01919-0 (PMC9724256; doi:10.1186/s12875-022-01919-0)
Supplement: Supplementary file 4 — Additional file 4: Appendix 4: Table A3. Description of full-text articles which discussed student-led healthcareservices as a strategy to improve PHC service delivery in ruralcommunities. [file 12875_2022_1919_MOESM4_ESM.docx]

Supplementary material Appendix 4, Table A3: Description of full-text articles which discussed student-led healthcare services as a strategy to improve PHC service delivery in rural communities

| Authors | Country | Article type | Findings |
| --- | --- | --- | --- |
| Atuyambe LM, et al, 2016 | Uganda | Research article | Students contribute meaningfully to primary health care delivery. Students are not only learning; they also contribute to delivery of health services at the facilities. Students were described as caring and compassionate, available on time and anytime, and as participating in patient care. They were willing to share their knowledge and skills, and stimulated discussion on work ethics. |
| Campbell DJ, et al, 2013 | Canada | Research article | Student-Run Clinics (SRCs) possess several unique features that may make them a potentially important primary healthcare resource for the homeless. Students are ideally suited to provide empathetic healthcare to this population. |
| Simpson SA and Long JA, 2007 | USA | Research article | Medical student-run health clinics offer myriad services to disadvantaged patients and are also a notable phenomenon in medical education. Wider considerations of community health and medical education should not neglect the local role of a student run health clinic. |
| Stuhlmiller CM and Tolchard B, 2019 | Australia | Research article | Student-led community health and wellbeing clinics offer free health services in a disadvantaged community, thereby improving overall health and wellbeing. The student-led clinic is an invaluable and sustainable link between students, health care professionals, community based organizations, the university, and the community. The community benefits from the clinic by learning to self-manage health and wellbeing issues. The benefits for students are that they gain practical experience in an interdisciplinary setting and through exposure to a community with unique and severe needs |
| Suen J, et al, 2020 | USA | Research article | As the need for health care services rise, alternative service delivery models such as student-led health interventions become attractive alternatives to alleviate the burden on healthcare. |
