# Supplementary material for: What improves access to primary healthcare services in rural communities? A systematic review
Source: BMC Prim Care. 2022 Dec 6;23:313. doi: 10.1186/s12875-022-01919-0 (PMC9724256; doi:10.1186/s12875-022-01919-0)
Supplement: Supplementary file 5 — Additional file 5: Appendix 5: Table A4. Descriptionof full-text articles which discussed outreach services or mobile clinics as astrategy to improve PHC service delivery in ruralcommunities. [file 12875_2022_1919_MOESM5_ESM.docx]

Supplementary material Appendix 5, Table A4: Description of full-text articles which discussed outreach services or mobile clinics as a strategy to improve PHC service delivery in rural communities

| Authors | Country | Article type | Findings |
| --- | --- | --- | --- |
| Bailey JJ, et al, 1994 | UK | Research article | Specialist outreach improves access healthcare services and shortens waiting times. |
| Bond M, et al, 2000 | UK | Research article | Outreach improves patient access to specialists. While the process of care is of higher quality in outreach than in outpatients, and the efficiency of care was also greater in the latter. |
| Bowling A, et al, 1997 | UK | Research article | The main advantages of outreach services are related to the greater convenience and better access to care for patients. |
| De Roodenbeke E, et al, 2011 | UK | Technical report | Outreach health services increases access and quality to health specialists in remote areas and connect different levels of the health system and strengthen the cooperation through a better utilization of the health workforce. These activities increase the effectiveness of front-line health workers and respond directly to patients’ concerns. |
| Gruen RL, et al, 2006 | Australia | Research article | Specialist outreach visits to remote disadvantaged communities improve access to specialist consultations and procedures without increasing elective referrals or demands for hospital inpatient services. |
| Gruen RL, et al, 2002 | Australia | Research article | Specialist outreach services overcome barriers relating to distance, communication, and cultural inappropriateness of services. Specialist outreach from a regional centre can provide a more equitable means of service delivery. |
| Gruen RL, et al, 2001 | Australia | Research article | Outreach is a more accessible, appropriate and efficient method of providing specialist medical services to remote communities. |
| Irani M, et al, 2007 | UK | Research article | Specialist outreach clinics demonstrate increased levels of patient satisfaction but may not be cost-effective. |
| Spencer N, 1993 | UK | Research article | Specialist outreach increases patient and professional choice and access to specialist consultant services, increases service flexibility, reduces unnecessary hospital visits, and enables more rational and relevant clinical decision making. |
| Aljasir B, et al, 2010 | Saudi Arabia | Research article | Mobile clinics provide healthcare to underserved populations and difficult-to-reach areas. Mobile clinics in rural areas can play a supporting role in the provision of healthcare services, and healthcare authorities can use such technologies to improve access and reduce the problem of travel on patients. |
| Cone PH, et al, 2016 | Haiti | Research article | Mobile clinics have been used successfully to provide healthcare services to people in hard-to-reach areas. Using mobile clinics among improves access to healthcare to medically underserved and vulnerable populations. |
| Diaz-Perez MD, et al, 2004 | Mexico | Research article | A community-based mobile clinic targeted toward rural immigrants can be effective in uncovering illness and in directing patients to a healthcare home. This is an important first step in eliminating health disparities among this population. |
| Edgerley LP, et al, 2007 | USA | Research article | Mobile health clinic has the potential to enhance access to the medical care system. It improves access to primary care for mothers and children. It helps women access care earlier in their pregnancies by helping to alleviate barriers such as transportation and cost. |
| Hill C, et al, 2012 | USA | Research article | Mobile health clinics are a promising tool to deliver proven cost-effective chronic disease prevention interventions in underserved areas and to meet the dual imperatives of controlling healthcare costs and reducing health disparities. |
| Lee EJ, et al, 1994 | USA | Research article | Mobile clinics provide quality healthcare that is accessible, available, and affordable. Mobile clinics provide cost effective health education, early detection of cases, and referral for low-income rural populations. With the diminishing quality of life and the difficulty in accessing healthcare in rural settings, this alternative model represents a new direction in PHC for low-income rural residents. |
| Neke NM, etal, 2018 | Tanzania | Research article | Mobile health clinics have improved coverage of essential maternal and child health (MCH) interventions. Reported are the increased engagement of the community and awareness of the importance of MCH services, which is believed to have a positive effect on uptake of services. Immunization, antenatal care, postnatal care and growth monitoring all seem to be successfully implemented in this mode of service delivery. However, this mode of service may face financial, human resource-related and logistic constraints. |
| Padmadas SS, et al, 2014 | Nepal | Research article | Mobile clinics significantly increase the uptake of vasectomy in hard-to-reach areas of Nepal. Reproductive health interventions should consider mobile clinics as an effective strategy to improve access to male-based modern methods and enhance gender equity in family planning. |
| Peters J, et al, 2013 | Nigeria | Research article | Mobile clinics are useful to improve the geographic accessibility of health services. Mobile clinic service offers a model of service delivery that has created high levels of user satisfaction in remote communities. |
