# Supplementary material for: What improves access to primary healthcare services in rural communities? A systematic review
Source: BMC Prim Care. 2022 Dec 6;23:313. doi: 10.1186/s12875-022-01919-0 (PMC9724256; doi:10.1186/s12875-022-01919-0)
Supplement: Supplementary file 6 — Additional file 6: Appendix 6: Table A5.Description of full-text articles which discussed family health program as astrategy to improve PHC service delivery in rural,communities. [file 12875_2022_1919_MOESM6_ESM.docx]

Supplementary material Appendix 6, Table A5: Description of full-text articles which discussed family health program as a strategy to improve PHC service delivery in rural, communities

| Authors | Country | Article type | Findings |
| --- | --- | --- | --- |
| Aquino R, et al, 2009 | Brazil | Research article | The FHP contributes toward reducing health inequalities. It has an important effect on reducing the infant mortality. |
| Dourado I, et al, 2011 | Brazil | Research article | The FHP has provided a new, more robust model of primary healthcare services designed to provide accessible, first contact, comprehensive, and whole person care that is coordinated with other healthcare services. It has positive results to improved availability, access to, and use of health services, and improved health indicators, such as reduced infant mortality. |
| Macinko J, et al, 2015 | Brazil | Perspective | FHP has evolved into a robust approach to providing primary care for defined populations by deploying interdisciplinary health care teams. The nucleus of each team includes a physician, a nurse, a nurse assistant and four to six full-time community health agents. FHP facilitated access and first-contact care are by locating healthcare teams near people’s homes. Lists of all residents in each geographic area permit delivery of longitudinal care or public health interventions, and each team is responsible for everyone in its catchment area. |
| Macinko J, et al, 2006 | Brazil | Research article | The FHP is associated with reduced infant mortality rate (IMR), suggesting it is an important, although not unique contributor to declining infant mortality. For example, in Brazil, A 10% increase in FHP coverage was associated with a 4.5% decrease in IMR |
