# Supplementary material for: What improves access to primary healthcare services in rural communities? A systematic review
Source: BMC Prim Care. 2022 Dec 6;23:313. doi: 10.1186/s12875-022-01919-0 (PMC9724256; doi:10.1186/s12875-022-01919-0)
Supplement: Supplementary file 7 — Additional file 7: Appendix 7: Table A6. Description of full-text articles whichdiscussed empanelment as a strategy to improve PHC service delivery in ruralcommunities. [file 12875_2022_1919_MOESM7_ESM.docx]

Supplementary material Appendix 7, Table A6: Description of full-text articles which discussed empanelment as a strategy to improve PHC service delivery in rural communities

| Authors | Country/  region | Article type | Findings |
| --- | --- | --- | --- |
| Bearden T, et al, 2019 | Global setting | Book | In many health care systems, empanelment is an important early step towards effective and coordinated PHC and can begin a paradigm shift from disease treatment to disease prevention. Empanelment enables PHC systems to move from reactive care oriented around visits, to proactive care that leverages the primary health care team’s potential to improve population health. |
| Chong P and Ee Tang W, 2018 | Global setting | Letter to editor | The empanelment of a patient to a team comprising family physicians, a nurse trained in chronic disease management and a care coordinator provide patients with longitudinal and interpersonal continuity of care, addressing a fundamental tenet of good primary care. Empanelment also fosters a provider-patient relationship over time, allowing the healthcare team to integrate a patients’ physical and mental health with social care issues in the comprehensive care of the patient. |
| McGough P, et al, 2018 | USA | Research article | A key strategy for population health is empanelment and panel management for patients in primary care. Empanelment is clearly identifying all the patients assigned to a primary care provider and their clinic. Panel management is identifying the care gaps for each ‘empaneled’ patient and creating plans to address those gaps. Empanelment identifies every primary care provider’s patient population for which they are accountable for improved health outcomes. |
| Primary health care performance initiative, 2019 | Global setting | Discussion paper | Empanelment (also referred to as population registration or rostering in some areas), a necessary aspect of primary care delivery, is an ongoing and deliberate set of actions to identify, match, and actively review and update data describing a group of people for whom a healthcare organization, care team, or provider is responsible. Additionally, both patients and providers are aware or their relationship. The listing is actively reviewed and regularly updated to ensure accuracy. |
