# Supplementary material for: What improves access to primary healthcare services in rural communities? A systematic review
Source: BMC Prim Care. 2022 Dec 6;23:313. doi: 10.1186/s12875-022-01919-0 (PMC9724256; doi:10.1186/s12875-022-01919-0)
Supplement: Supplementary file 8 — Additional file 8: Appendix 9: Table A8.Description of full-text articles which discussed telemedicine or mobile healthas a strategy to improve PHC service delivery in ruralcommunities. [file 12875_2022_1919_MOESM8_ESM.docx]

1. Supplementary material Appendix 8, Table A7: Description of full-text articles which discussed community health funding schemes as a strategy to improve PHC service delivery in rural communities

| Authors | Country/  region | Article type | Findings |
| --- | --- | --- | --- |
| Atnafu DD, et al, 2018 | Ethiopia | Research article | This study found that there was a significant difference in the rate of healthcare utilization between insured (50.5%) and uninsured (29.3%) households, suggesting that utilization of health services among insured households with community-based health insurance was higher. |
| Balamiento NC, et al, 2018 | Philippines | Thesis | Findings of this study reveal that participation in social health insurance is robustly and positively correlated with inpatient and outpatient care utilization for both adults and children under 15 years of age. |
| Blanchet  NJ, et al, 2012 | Ghana | Research article | This study reported that on average individuals enrolled in the insurance scheme are significantly more likely to obtain prescriptions, visit clinics and seek formal health care when sick. This suggests that suggest that community health insurance plays a role to increase access to the formal health care sectors. |
| [Farrell](https://ajph.aphapublications.org/author/Farrell%2C+Caitlin+M) CM and [Gottlieb](https://ajph.aphapublications.org/author/Gottlieb%2C+Aaron) A, 2020 | USA | Research article | Health insurance coverage was associated with increased utilization of outpatient, inpatient, and emergency department health care among justice-involved individuals. Therefore, expanding access to health insurance in this population has the potential to increase care utilization of all types and decrease barriers to medical services. |
| Jütting J, 2001 | Senegal | Research article | This study showed that access to health insurance can have a positive impact on the economic and social situation of their members. To enlarge the access to health care of the poor and the rural population, community based health insurance schemes can be an important element and a first step. It allows to a limited degree to pool risks and thereby leads to an improvement in the health care system. |
| Mwaura JW, et al, 2012 | Kenya | Research article | This study found that insured members reported higher use of hospitalization care than the non-insured. This confirmed that prepayment schemes and the pooling of risk could reduce financial barriers to health care among the urban poor. |
| Nshakira‑Rukundo, et al, 2021 | Uganda | Research article | This study reported that community-based health insurance enrolment increased the probability of using long-lasting mosquito nets by 26% (84% points of the control group) and deworming by 18% (29% points). |
| Thi Thu Thuong N, 2020 | Vietnam | Research article | This study showed that health insurance policy increased the number of outpatient and inpatient visits for the enrolled participants in Vietnam and this suggested that health insurance coverage increases access to healthcare services for citizens. |
| USAID, 2011 | Ethiopia | Report | This report showed that health service utilization in Ethiopia is increasing due to improved access to health services. The increased and improved cash flow has had a positive effect on the availability of drugs and other supplies, which in turn has improved the quality of health services the facilities provide. |
| Wang W, et al, 2014 | Africa | Research article | This study found significant positive effects of health insurance coverage on at least one measure of maternal health care use. The positive impact of health insurance appeared more consistent on the use of facility-based delivery than use of antenatal care. This study provides clear evidence that health insurance has contributed to the increased use of maternal health care services. |
| William C. Hsiao, 2001 | Global setting | Discussion paper | For low-income countries, community health financing has modest ability to increase the total amount of funds for healthcare. Properly structured community health financing system can significantly improve efficiency, reduce the cost of health care, improve quality and health outcomes, and pool risks. Community-financing schemes could improve preventive services and reduce the incidence of disease. It could also improve people’s access to health care and the quality of services, thus improving their health status. Community health financing could also improve risk pooling and reduce health-induced impoverishment |
