# Supplementary material for: What improves access to primary healthcare services in rural communities? A systematic review
Source: BMC Prim Care. 2022 Dec 6;23:313. doi: 10.1186/s12875-022-01919-0 (PMC9724256; doi:10.1186/s12875-022-01919-0)
Supplement: Supplementary file 9 — Additional file 9: Appendix 8: Table A7.Description of full-text articles which discussed community health funding schemes as a strategy to improve PHC service delivery in ruralcommunities. [file 12875_2022_1919_MOESM9_ESM.docx]

Supplementary material Appendix 9, Table A8: Description of full-text articles which discussed telemedicine or mobile health as a strategy to improve PHC service delivery in rural communities

| Authors | Country/  region | Article type | Findings |
| --- | --- | --- | --- |
| Anstey Watkins JOT, et al, 2018 | South Africa | Research article | The use of mobile phones in poor, remote rural areas provides opportunities to improve healthcare access and delivery. Mobile phones have been evolving to fill the gaps to augment primary care services. |
| Bashshur RL, et al, 2009 | USA | Research article | Telemedicine improves access to primary, secondary, and tertiary healthcare for a wide range of conditions; promotes patient-centered care at lower cost and in local environments that also contributes to stabilizing local healthcare; and promotes individual adoption of healthy lifestyles and self-care. |
| Buckwalter KC, et al, 2002 | Global setting | Research article | Technology-based delivery methods, such as videophone skill training, and one- and two-way interactive computer networks, are envisioned as contributors for improving rural residents’ access to services, individualizing rural health care, increasing rural health practitioners’ continuing education opportunities, and improving quality and cost-efficiency of care. |
| Custodio R, et al, 2009 | USA | Research article | Telemedicine can increase healthcare access, improve care delivery systems, engage in culturally competent outreach and education, and enhance workforce development and training. |
| Kuntalp M, et al, 2004 | Global setting | Research article | A simple and low-cost Internet-based tele-consultation system could effectively solve the healthcare access problems in underserved areas of developing countries. |
| Marcin JP,et al, 2004 | USA | Research article | Subspecialty telemedicine consultations can be provided to rural communities, medically underserved communities. Telemedicine should be considered as a means of facilitating care. It is more accessible, family-centered, and coordinated among patients and their healthcare providers. |
| Meier CA, et al, 2013 | Global setting | Research article | eHealth holds the promise of revolutionizing healthcare by improving its efficiency; extending and enhancing its reach; energizing and engaging its practitioners and their patients; and in the process, democratizing, decentralizing, and even partially demystifying the practice of medicine. |
| Norton SA, et al, 1997 | USA | Research article | Telemedicine will have a revolutionary effect on both public and private healthcare systems in the areas of cost savings and increased access. It is a useful way to provide healthcare services to remote or underserved communities. |
| Olu O, et al, 2019 | Global setting | Perspective | Digital health has several benefits. These include; improving access to health care services especially for those in hard-to-reach areas, improvements in safety and quality of healthcare services and products, improved knowledge and access of health workers and communities to health information; cost savings and efficiencies in health services delivery; and improvements in access to the social, economic and environmental determinants of health. |
| Price M, et al, 2013 | Global setting | Research article | Effective use of mobile applications has the potential to increase access to evidence-based care; better inform consumers of care; increase the use of evidence-based practices; and enhance care after formal treatment. |
| Raza T, et al, 2009 | USA | Research article | Telemedicine is one of the solutions for rural subspecialty healthcare delivery. The provision of subspecialty services using telemedicine to a remote and underserved population improves access to subspecialty care. |
| Ryan MH, et al, 2016 | USA | Research article | Given the difficulties faced by low income and medically underserved communities in accessing healthcare services, the use of IT tools may improve their’ access to health information in ways that could enhance patient knowledge and self-management, and perhaps positively impact health outcomes. |
| Shouneez YH, et al, 2015 | South Africa | Dissertation | mHealth strengthens community-based diseases screening programs. For instance, the hearScreen smart phone is useful in the hearing screening program and offers benefits such as automated test protocols and interpretation, integrated noise monitoring, data capturing and data sharing. |
