# Supplementary material for: What improves access to primary healthcare services in rural communities? A systematic review
Source: BMC Prim Care. 2022 Dec 6;23:313. doi: 10.1186/s12875-022-01919-0 (PMC9724256; doi:10.1186/s12875-022-01919-0)
Supplement: Supplementary file 10 — Additional file 10: Appendix 10: Table A9.Description of full-text articles which discussed promoting the role of workingwith traditional healers as a strategy toimprove PHC service delivery in rural communities. [file 12875_2022_1919_MOESM10_ESM.docx]

Supplementary material Appendix 10, Table A9: Description of full-text articles which discussed promoting the role of working with traditional healers as a strategy to improve PHC service delivery in rural communities

| Authors | Country/  region | Article type | Findings |
| --- | --- | --- | --- |
| Habtom GK, 2015 | Eritrea | Research article | Selective integration of traditional medical practice with the primary healthcare system is important since most rural communities used traditional medicine and self-care for the treatment of serious illnesses, irrespective of availability of western medical service in many parts of Eritrea. |
| Kang’ethe SM,2009 | Botswana | Research article | Traditional healers are important players in care giving of persons with various ailments. |
| Mbwambo ZH, et al, 2007 | Tanzania | Research article | TM has had cultural acceptance in the majority of people and has the potential to solve healthcare problems. Incorporating traditional healers into public health delivery addresses the healthcare needs of people with limited access to allopathic medicine. |
| Mishra SR, et al, 2014 | Nepal | Research article | Complementary and alternative medicine has been a part of human life and practices since the beginning of time. The role of complementary and alternative medicine to promote health is undisputed particularly in light of its role in health promotion and well-being. |
| Payyappallimana U, 2009 | Global setting | Research article | Knowledge about TM has a catalyzing effect in meeting health sector development objectives and will continue to be so in both the developed and developing worlds. |
| Poudyal AK, et al, 2003 | Nepal | Research article | Traditional healers have a better knowledge of allopathic medicine, practiced modern treatment using first aid kits, and were more likely to refer patients to government health workers. |
| WHO, 2000 | Africa | Technical paper | TM and its practitioners play in health development in Africa. Integration of TM in health system will result in increased coverage and access to, healthcare. The use of TM of proven efficacy and safety can supplement other efforts to achieve health for all. |
