# Supplementary material for: What improves access to primary healthcare services in rural communities? A systematic review
Source: BMC Prim Care. 2022 Dec 6;23:313. doi: 10.1186/s12875-022-01919-0 (PMC9724256; doi:10.1186/s12875-022-01919-0)
Supplement: Supplementary file 11 — Additional file 11: Appendix 11: Table A10.Description of full-text articles which discussed working with non-profitprivate sectors and non-governmental organizations as a strategy to improve PHC service delivery in rural communities. [file 12875_2022_1919_MOESM11_ESM.docx]

Supplementary material Appendix 11, Table A10: Description of full-text articles which discussed working with non-profit private sectors and non-governmental organizations as a strategy to improve PHC service delivery in rural communities

| Authors | Country/  region | Article type | Findings |
| --- | --- | --- | --- |
| Ahmed N, et al, 2019 | Sudan | Research article | Private health providers of immunization services have especially been critical in filling the gaps in government services in hard-to-reach or conflict-affected areas and among marginalized populations in Sudan, and thus in reducing inequities in access. |
| Baqui AH, et al, 2008 | India | Research articles | NGO facilitation of government programmes in India is a feasible strategy to improve equity of maternal and neonatal health programmes. Improvements in equity were most pronounced for household practices, and inequities were still apparent in health care utilization. |
| Biermann O, et al, 2016 | Ecuador | Research article | Non-governmental organizations (NGOs) have a key role in improving health in low- and middle-income countries. Their work needs to be synergistic, complementary to public services, and rooted in community mobilization and collective action. The population perceives the NGO positively, linking it to healthcare improvements. The presence of an NGO in the operation, however, may contribute to unrealistic expectations of health services, affecting perceptions of the latter negatively. |
| Edimond BJ, 2011 | South Sudan | Dissertation | The study revealed that that NGOs have made significant contributions to reproductive and sexual health in many areas in south Sudan. The research also found that effectively run NGO reproductive and child health services have decreased child and maternal mortality. The importance of NGOs in the delivery of services have gained recognition in terms of filling gaps in government programmes. |
| Ejaz I, et al, 2011 | Pakistan | Research article | Involving the non-governmental (NGOs) for health system strengthening eventually contributes to create a healthcare system reflecting an increased efficiency, more equity and good governance in health. International and local NGOs have endeavored to fill the gaps in health service delivery, research and advocacy. NGOs have relatively performed better and achieved the results because of the flexible planning and the ability to design population based projects on health education, health promotion, social marketing, community development and advocacy. |
| Feng Shi WU, 2005 | China | Commentary | International non-governmental organizations were among the first international actors that responded to the emergence of AIDS crisis in China. Despite their organizational differences, most of these non-governmental actors present the characteristics of independent mission, localized practice and diverse working focus. Even though they are constrained by financial and other factors compared with multilateral and bilateral official assistance agencies, they have still played a unique role in fighting against AIDS in China as technical experts, public educators, and civil society supporters. |
| Mercer A, et al, 2004 | Bangladesh | Research article | NGOs can play an important role in health service delivery in rural Bangladesh. Bangladesh Population and Health Consortium (BPHC) has demonstrated that an NGO programme can provide Essential Services Package (ESP) effectively to a large, widely distributed rural population and achieve high coverage and relatively good health outcomes. The management information system (MIS) has provided evidence to support the view that local NGOs are able to reach poor women and children whose access to government or profitmaking services is restricted. This study also reported that relatively high coverage has been achieved for reproductive and child health services, as well as lower infant and child mortality. |
| Ricca J, et al, 2013 | Africa, Asia and Caribbean. | Research article | NGO projects implementing community-based intervention packages appear to be effective in reducing child mortality in diverse settings. There is plausible evidence that they raised coverage for a variety of high-impact interventions and improved under five-year mortality rate by more than twice the concurrent secular trend. |
| Bandy G,et al, 2008 | Global setting | Technical report | FBOs offer compassionate care to people in need. The inclusion of faith-based organizations in the PHC system adds greater potential for breadth and effectiveness of care. Faith-based organizations deliver treatment, care and prevention activities in accordance with WHO strategic priorities and PHC principles. |
| Chand S, et al, 2007 | Global setting | Technical report | Faith-based healthcare facilities provide a significant percentage of healthcare services. With networks that reach even the most remote communities, many FBOs are well positioned to promote demand and access for healthcare services. Partnerships among FBOs are critical in promoting, delivering and increasing quality, quantity and access of services, and ensure sustainability by influencing behaviors at community, family and individual level. |
| Green A, et al, 2002 | SSA | Discussion paper | FBOs play major roles in healthcare delivery in sub-Saharan Africa. The combined forces of FBOs and governments can achieve the objectives of universally available and affordable healthcare. |
| Levin J, 2014 | Global setting | Research article | FBOs represent a long standing resource for health promotion and disease prevention. Formal partnership between the faith-based and public health sectors encompasses activities in the fields of health behavior and health education, health policy and management, and environmental health. |
| Magezi V, 2018 | East Africa | Research article | The role of FBOs in health care is ranged from establishing and managing large hospitals to rural communities. FBOs through formal and informal programs involve in health promotion. |
| Villatoro AP, et al, 2016 | USA | Research article | FBOs play an integral role in the healthcare system by increasing health seeking behaviors, providing religion-based health services, and delivering supportive services that address common access barriers. FBOs offer unique opportunities to address some of the cultural barriers. |
| Zahnd WS, et al, 2018 | USA | Commentary | Faith- based interventions have been an effective approach to improving cancer screening among rural and underserved populations. Similarly, faith community nurses (FCNs) may be an effective agents for implementing evidence- based cancer screening strategies in rural communities. |
